# Psychometric properties of a brief measure of autonomy support in breast cancer patients

**DOI:** 10.1186/s12911-015-0172-4

**Published:** 2015-07-09

**Authors:** Dean Shumway, Kent A. Griffith, Reshma Jagsi, Sheryl G. Gabram, Geoffrey C. Williams, Ken Resnicow

**Affiliations:** University of Michigan, School of Medicine, 2800 Plymouth Road, Ann Arbor, MI 48109 USA; Emory University, School of Medicine, 69 Jesse Hill Jr. Drive SE, Atlanta, GA 30303 USA; University of Rochester, School of Medicine, 500 Joseph C. Wilson Blvd, Rochester, NY 14611 USA; University of Michigan, School of Public Health, 109 Observatory Street, Ann Arbor, MI 48109-2029 USA

**Keywords:** Autonomy support, Breast cancer, Health Care Climate Questionnaire

## Abstract

**Background:**

The *Health Care Climate Questionnaire* measures patient perceptions of their clinician’s autonomy supportive communication. We sought to evaluate the psychometric properties of a modified brief version of the *Health Care Climate Questionnaire* (mHCCQ) adapted for breast cancer patients.

**Methods:**

We surveyed 235 women aged 20–79 diagnosed with breast cancer within the previous 18 months at two cancer specialty centers using a print questionnaire. Patients completed the mHCCQ for their surgeon, medical oncologist, and radiation oncologist separately, as well as the overall treatment experience. Exploratory factor analysis (EFA) using principal components was used to explore the factor structure.

**Results:**

One hundred sixty out of 235 (68.1 %) women completed the survey. Mean age was 57 years and time since diagnosis was 12.6 months. For surgeon, medical oncologist, and radiation oncologist ratings separately, as well as overall treatment, women rated 6 dimensions of perceived physician autonomy support. Exploratory factor analysis indicated a single factor solution for each clinician type and for the overall experience. Further, all six items were retained in each clinician subscore. Internal consistency was 0.93, 0.94, 0.97, and 0.92 for the overall, surgeon, medical oncologist, and radiation oncologist scales, respectively. Hierarchical factor analysis demonstrated that a summary score of the overall treatment experience accounts for only 52 % of the total variance observed in ratings of autonomy support for the three provider types.

**Conclusions:**

These results describe the first use of the mHCCQ in cancer patients. Ratings of the overall treatment experience account for only half of the variance in ratings of autonomy support, suggesting that patients perceive and report differences in communication across provider types. Future research is needed to evaluate the relationship between physician communication practices and the quality of decision making, as well as other outcomes among cancer patients.

## Background

Patient-centered physician communication plays a central role in ameliorating the psychological burden associated with a new cancer diagnosis and may have a positive impact on patient health outcomes [1]. After diagnosis, breast cancer patients quickly encounter numerous complex decisions that add to the emotional burden of anxiety, uncertainty, and fear. The physician’s approach to exchanging information and making decisions can significantly impact the cancer experience. For example, patients who were given more decisional control in the choice of mastectomy versus breast conserving surgery reported less depression, anxiety, and psychological morbidity, as well as higher levels of quality of life [2–6]. Patient satisfaction and recall of information is also closely related to the quality of doctor-patient communication during the initial oncology consultation [7, 8].

Physician communication practices also exert a significant influence on patient compliance. An autonomy supportive style—where physicians offer choice, provide a meaningful rationale, minimize pressure, support patient input, and acknowledge the patient's feelings and perspectives—is an essential element for facilitating internalization of health behavior, according to Self-Determination Theory [9]. Internalization refers to the process whereby a behavior that was initially prompted by external sources is regulated with an experience of autonomy and an accompanying sense of competence [10, 11]. In other words, patients perceive themselves to be autonomous when their behavior is experienced with a sense of volition and choice, whereas patients perceive themselves to be controlled when they experience pressure or coercion to think, feel, or behave in certain ways [11]. Internalization of a treatment decision as one’s own, rather than being acted upon by an external influence, has important effects on patients’ coping, cooperation, and satisfaction with care [12].

The *Health Care Climate Questionnaire* has been used to measure how the quality of a physicians’ autonomy support (as reported by patients) influences motivation, behavior change, and well-being [13]. It is based on principles of self-determination theory, with the hypothesis that autonomy-supportive communication by providers facilitates more self-determined motivation and perceived competence, and thereby more lasting behavioral change. Higher levels of autonomy support, in contrast to a controlling communication style, have been shown to positively influence compliance and success of self-regulated behavior, such as smoking cessation, weight loss, glucose control, and medication compliance [9, 11, 14–18]. The original *Health Care Climate Questionnaire* contained 15 items and was shorted to the *modified Health Care Climate Questionnaire* (mHCCQ) to reduce item redundancy while retaining a full representation of the concept of autonomy support [15]. The items retained were those with higher factor loading in previous studies, and were recommended by the developer of the scale (GW, personal communication).

Several other standardized measures of patient-centered communication have been described, such as the Behavior Change Counseling Index (BECCI) [19] and the Motivational Interviewing Treatment Integrity Scale [20]. Although these instruments have been described in contexts in which the purpose is to change health behaviors, they have been designed primarily for trained raters to assess physician communication practices, rather than for evaluation from the patient perspective. Little is known regarding how cancer patients’ perceived autonomy support of their physicians may influence decision-making, quality of life, and satisfaction. We therefore sought to explore the use of the mHCCQ in breast cancer patients.

The primary aim of this study was to explore the psychometric properties of the mHCCQ, and to evaluate its use in breast cancer patients. We also sought to determine whether patients differentiate the perceived autonomy support from surgery, medical oncology, and radiation oncology providers or if they perceive the multidisciplinary group of cancer care providers as a single entity. The goal was to determine if the short form rating of overall experience would suffice for evaluation of autonomy support in this context, or if a significant amount of variance would be missed by failing to evaluate the same measures in each individual specialty.

## Methods

### Study Sample and Data Collection

The target sample was women aged 20–79 who were diagnosed withbreast cancer within the previous 18 months. The sample included patients with American Joint Committee on Cancer (AJCC) stage 0 – III breast cancer, with the intention to evaluate the full spectrum of experiences in patients who might have many treatment options, as well as those with more advanced disease in whom treatment recommendations might be more prescriptive. Participants were recruited from Memorial Sloan-Kettering Cancer Center (MSKCC) in New York and from Emory University Hospital Midtown, the Winship Cancer Institute of Emory University, and Grady Memorial Hospital in Georgia between June and September 2013. Based on an estimated sample size for adequacy of psychometric analyses, we set a quota sample of 200 completed surveys.

At MSKCC, eligible breast cancer patients were approached in clinic and asked to complete the survey. Patients who met the inclusion criteria were identified by examining the clinic schedule for the upcoming day and approaching all eligible patients on a selected day. These women were given the option to take the survey home to complete if requested. A $10 incentive was provided to respondents upon completion of their survey.

At the Georgia sites, eligible breast cancer patients were identified by clinic records and mailed a survey packet which included a $10 pre-incentive. The institutional review boards of the University of Michigan, MSKCC, and Emory University approved all study procedures and materials.

The response rate was 83.8 % (93 of 111) in New York and 54.0 % (67 of 124) in Georgia, for a combined response rate of 67.2 % (158/235). For factor and internal consistency analysis by physician specialty, which required all items to be completed, the final analytical sample size was 155, 157, 138, and 106, for the overall treatment experience (hereafter referred to as “overall”), surgeon, medical oncologist, and radiation oncologist scales, respectively. For hierarchical factor analysis that required all provider-specific items, the analytical sample size was 106.

### Measures

#### Patient characteristics

Participants were asked about their age, race, ethnicity, and level of education as well as the amount of time (in months) since their breast cancer diagnosis. We also asked yes/no questions to ascertain whether or not they had received various treatments, specifically lumpectomy, mastectomy, radiation therapy, and chemotherapy, and whether they experienced moderate or severe toxicity during their treatment (defined as nausea, vomiting, diarrhea, shortness of breath, pain, or arm swelling).

Perceived autonomy support was assessed with six questions that measured patients’ perceptions of the degree to which their physicians were autonomy supportive. Patients responded to the six questions for their overall treatment experience, followed by questions about their surgeon, medical oncologist, and radiation oncologist, in that order. The six questions, which were provided by the scale’s developer (GW) were asked as follows:

I feel that my (insert breast cancer treatment doctors, surgeon, medical oncologist, or radiation oncologist)…

1. …provided me with choices and options for my breast cancer treatment.
2. …understood how I saw things with respect to my breast cancer.
3. …expressed confidence in my ability to make decisions.
4. …listened to how I would like to handle my breast cancer treatment.
5. …encouraged me to ask questions.
6. …tried to understand how I saw things before offering an opinion.

Responses were on a 7 point scale anchored with: not at all true (1), somewhat true (4), and very true (7).

### Analyses

Exploratory factor analysis (EFA) using principal components was used to explore the factor structure, i.e. the number of underlying constructs measured by the items. We began by retaining factors with Eigenvalues near to 1 (indicating that approximately 16.7 % of the variance was explained) and required item-loadings of > 0.45 as indication that the items should be retained. After scales were formed from the factor(s), we measured their internal consistency using Cronbach’s alpha and reported the correlation between the scales as calculated for the four groups (3 provider groups and the overall rating) assessed using Spearman’s rank coefficient. We also evaluated the correlation between provider level scales and the overall scale. We then explored the association of scales stratified by the surgery, chemotherapy, and radiation received, and by patient characteristics using the Kruskal-Wallis test. When the Kruskal-Wallis test suggested a significance difference among the groups, pairwise Wilcoxon Rank-sum tests were performed.

In order to explore whether assessment was needed on the provider level rather than simply asking about overall treatment experience, we examined inter-item correlations for 18 items measured at the provider-specific level using Spearman’s rank coefficient and hierarchical EFA with first- and second-order factors. The first order EFA was used to determine the common constructs/factors measured by the 18 items. It was hypothesized if patients did differentiate between providers that the 6 items asked about each specific provider would compose common factors, with three common factors, one for each provider type discovered. Conversely, if patients did not differentiate between providers, then it was hypothesized that the same questions asked across the three provider types would compose common factors, resulting in 6 factors, each with three items. Once the first-order factor solution was decided, those factor solutions were then used as the inputs into the second-order EFA. If patients did not differentiate between provider types, it was hypothesized that a single common second-order factor would explain the majority of the variance of the provider-level first-order factors. If, however, patients did differentiate between providers, a single common second-order factor would leave considerable variance unexplained. Additionally the feasibility of the single second-order factor was determined by considering the interpretability of the first-order factors loadings. The SAS System version 9.3 (SAS Institute Inc., Cary, NC, USA) was used for all statistical analyses. For statistical tests, p-values at or below 5 % were considered significant.

## Results

157 women completed the survey at an average of 12.6 months after diagnosis. Mean age was 56.9 years. The sample was 80.4 % white, 13.7 % black, 3.9 % Asian, and 4.6 % Hispanic. Most (87.8 %) had some college education or greater. The majority underwent lumpectomy (62.6 %) and radiation therapy (68.4 %), with 38.1 % undergoing mastectomy and 41.8 % receiving chemotherapy (Table 1).

**Table 1:** Sample description

	Mean/N	Range/%
Age (mean (range))	56.9	(25–79)
Months since diagnosis (mean (range))	12.6	(2–60)
Race/Ethnicity (%)		
White	120	78.4
Black	21	13.7
Asian	6	3.9
Hispanic	6	3.9
Missing	5	
Education (%)		
HS or less	20	12.8
Some college	29	18.6
College graduate	40	25.6
Post-graduate degree	67	43.0
Missing	2	
Treatment Received (%)		
Radiation therapy	106	67.5
Chemotherapy	64	41.3
Lumpectomy	97	62.6
Mastectomy	59	38.1

For overall ratings of autonomy support, as well as for provider specific ratings (surgeon, medical oncologist, and radiation oncologist), EFA for each indicated a single factor solution. For overall treatment experience and provider specific scales, only one Eigenvalue was greater than 1.0, and in each case the percentage of variance explained by the first factor was above 70 % (range 73.2 % to 86.7 %). Factor loadings for each of the 6 questions onto the first factor were uniformly high, all above 0.7, meeting our criteria for inclusion (factor loading >0.45) and were consistent across all 4 categories (Table 2). Internal consistency assessed by Cronbach’s alpha was 0.93, 0.94, 0.97, and 0.92 for the overall, surgeon, medical oncologist, and radiation oncologist scales using all 6 items, as shown in Table 3.

**Table 2:** Factor loadings for autonomy support

	Factor Loadings			
Item	Overall (*n* = 155)	Surgeons (*n* = 157)	Medical Oncologist (*n* = 138)	Radiation Oncologist (*n* = 106)
Provided me with choices	0.94	0.94	0.95	0.96
Understood how I saw things	0.93	0.92	0.94	0.92
Expressed confidence	0.90	0.92	0.94	0.90
Listened	0.86	0.92	0.94	0.88
Encouraged questions	0.75	0.85	0.93	0.84
Tried to understand	0.74	0.75	0.88	0.76

**Table 3:** Internal consistency (alpha) for four autonomy support scales

Target	Overall (*n* = 155)	Surgeon (*n* = 157)	Medical Oncologist (*n* = 138)	Radiation Oncologist (*n* = 106)
Alpha with all items	0.93	0.94	0.97	0.92
Alpha if item removed				
Provided me with choices	0.93	0.94	0.96	0.93
Understood how I saw things	0.92	0.93	0.96	0.90
Expressed confidence	0.92	0.92	0.96	0.90
Listened	0.91	0.92	0.96	0.89
Encouraged questions	0.94	0.95	0.97	0.93
Tried to understand	0.91	0.93	0.96	0.89

Mean scale scores demonstrated significant differences between the level of education and perceived autonomy support for the overall, surgeon, and medical oncologist scales (Table 4), with the appearance of a trend toward an inverse relationship with the education level across the scales. Black patients reported significantly higher mean scores than Asian patients in the overall and medical oncologist categories. Women who underwent lumpectomy reported higher perceived autonomy support from their medical oncologist than women who underwent mastectomy. Age, time since diagnosis, and receipt of chemotherapy or radiation were not related to scale scores. There were no significant differences in ratings of autonomy support for patients who experienced moderate or severe toxicity.

**Table 4:** Scale means by patient demographic and health factors

	Overall		Surgeon		Medical Oncologist		Radiation Oncologist	
	N	Mean	N	Mean	N	Mean	N	Mean
Scale Mean	155	6.3	157	6.4	138	6.0	106	6.1
Age								
<57	74	6.4	76	6.5	69	6.0	48	6.3
≥57	80	6.3	80	6.4	68	5.9	57	5.9
Months since Diagnosis								
<12	60	6.3	61	6.5	51	5.7	38	6.2
> =12	94	6.3	95	6.4	86	6.2	67	6.0
Race/Ethnicity								
White	123	6.3	125	6.4	108	5.8^B^	80	6.1
Black	22	6.7^A^	22	6.6	20	6.7^AB^	19	6.3
Asian	6	5.7^A^	6	6.5	6	5.9^A^	3	5.5
Education								
HS or less	19	6.8^AB^	20	6.8^A^	19	6.6^AB^	14	6.4
Some college	29	6.6^C^	29	6.4	27	6.4^CD^	23	6.2
College graduate	39	6.3^A^	40	6.6^B^	34	5.6^AC^	26	6.4
Post-graduate degree	67	6.1^BC^	67	6.2^AB^	57	5.8^BD^	42	5.7
Treatment Received								
Radiation therapy								
Yes	106	6.4	106	6.5	98	6.1	101	6.1
No	49	6.2	51	6.3	40	5.8	5	5.9
Chemotherapy								
Yes	64	6.3	64	6.3	64	6.3	42	6.2
No	89	6.3	91	6.5	72	5.8	62	6.1
Lumpectomy								
Yes	114	6.4	115	6.4	100	6.2^A^	95	6.1
No	38	6.2	38	6.4	36	5.4^A^	10	6.3
Mastectomy								
Yes	57	6.3	59	6.3	52	5.8^A^	19	6.1
No	95	6.4	95	6.5	83	6.2^A^	84	6.2
Moderate or Severe Toxicity								
Yes	134	6.3	136	6.4	117	5.9	93	6.1
No	21	6.4	21	6.5	21	6.4	13	6.1

When the Kruskal-Wallis test p value was <0.05,  pair-wise comparisons (without multiplicity correction) were conducted with significant differences (*p* < 0.05) between groups marked by like superscripts

In order to determine whether the variance in ratings of autonomy support as measured separately for the provider types can be explained by a summary assessment of the providers overall, hierarchical EFA was conducted, beginning with analysis of the provider-level items (6 items from each specialty for a total of 18 items). This first-order EFA yielded a three-factor solution that explained 80.7 % of the item total variance with the first three factors explaining 48.8 %, 17.5 %, and 14.4 % of total variance, respectively. The first factor had dominant factor loadings for the 6 medical oncologist items, the second for surgeon-related items, and the third factor for radiation oncologist items (Table 5), supporting our hypothesis that patients differentiate between providers. When these three factors were used as the inputs for the second-order EFA, each of the three first-order factors loaded nearly equally (0.68 to 0.74) onto the single second-order factor and explained 52.2 % of the first-order factor variance. The second-order factor solution was significantly correlated with the scale assessment for the overall treatment experience, with Spearman’s rank correlation coefficient of 0.75, suggesting that the second-order factor is indeed measuring an overall assessment of autonomy support. These results further support our hypothesis that patients do differentiate between the level of perceived autonomy support from each provider type, as the single second-order factor (which is representative of a summary score of the overall treatment experience) left nearly half the variance of the first-order factors unexplained.

**Table 5:** Factor loadings for second-order single factor solution

Items	Second-Order Factor
Factor 1 – Medical Oncologist dominated factor	0.74
Factor 2 – Surgeon dominated factor	0.74
Factor 3 – Radiation Oncologist dominated factor	0.69

The scales for each provider category were significantly positively correlated with the overall scale, at 0.74, 0.64, and 0.61 for the surgeon, medical oncologist, and radiation oncologist, respectively. However, provider-level scales were less positively inter-correlated (0.47 to 0.55). As shown in Table 4, mean scale scores for the four categories were 6.3, 6.4, 6.0, and 6.1 (out of 7) for overall, surgeon, medical oncologist, and radiation oncologist, respectively.

## Discussion

The primary finding from these analyses is that perceptions of autonomy supportive communication in breast cancer patients are consistently comprised of six dimensions for surgeons, medical oncologists, and radiation oncologists, as well as for the aggregate treatment experience. For patient perceptions of provider communication assessed using the mHCCQ, EFA for each clinician type yielded a single factor solution. Each of the 6 items loaded uniformly onto the single factor and demonstrated high internal consistency. These results increase our confidence that the six items on the mHCCQ are measuring a single underlying dimension for how breast cancer patients perceive the autonomy supportive communication style of their physicians.

A second finding from these analyses is that ratings of the overall treatment experience account for only half of the variance, suggesting that patients perceive and report differences in communication across provider types. When the three treatment-specific scales were grouped together for hierarchical EFA (6 items from each specialty for a total of 18 items), it is interesting to note that the three-factor solution loaded nearly equally onto a second-order factor, and that each of the three factors held together according to each specialty, rather than by individual questions. This suggests that answers to each question are more highly correlated with other questions on the same physician, rather than for the same question across provider types. Analysis of inter-item correlation across the 3 provider types yielded similar results, with higher correlation with other questions about each provider type than to the identical question asked of other specialties (data not shown). While it is possible that this may partially be related to grouping of provider questions together in sequence on the survey, it appears that patients clearly tend to differentiate their ratings of autonomy support according to each provider, rather than lumping their experience into an overall rating. Therefore, while assessment of only the aggregate treatment experience provides a general sense of how patients perceive providers’ autonomy supportive communication practices, assessing specialist-specific ratings appears to yield a more granular picture of patient experience. In situations where questionnaire length is limited or respondent burden is a particular concern, measuring only the overall score may be appropriate.

The correlation of specialty-specific scores with overall scores was slightly stronger for surgeons than for medical oncologists and radiation oncologists. This may indicate that the surgeon contributes more to patients’ overall treatment appraisal than the medical oncologist or radiation oncologist. However, on hierarchical factor analysis, we found that the second order factor, interpreted as a correlate of the overall treatment experience, was driven nearly equally by ratings of the surgeon and medical oncologist, with a lesser contribution from the radiation oncologist. It is also possible that because surgeon items were placed before medical oncology and radiation oncology items on the survey, an ordering effect contributed to the observed correlation. We previously observed a small ordering effect for when the overall experience and provider-specific experiences were rated (Resnicow et al., under review). Studies using random ordering of all items may be useful to determine the full impact of ordering on subject responses.

The overall means of the four scales were high at 6.0 or higher (out of 7). This positively skewed distribution is slightly higher than what has been reported in other settings using the mHCCQ [13], and is consistent with numerous prior studies of communication and decision-related ratings in cancer patients demonstrating high patient-reported satisfaction [21–24]. These high scores may in part be inflated by patient response bias related to the desire to feel good about their treatment experience in order to minimize dissonance [25]. With an average time from diagnosis of 12.6 months, recall bias and forgetting may have influenced responses. Both survey sites are renowned cancer centers, which could have biased scores upward as well. Given our study sample, we are unable to determine if surveying patients from less specialized treatment centers would have resulted in lower scores. As a measure of subjective perceptions of autonomy support, the values observed in our data may be considered inherently valid. Further external validity would be demonstrated if higher scores predicted increase in autonomous self-regulation (i.e. internalization), relative intrinsic aspirations, perceived competence, vitality, and compliance with lifestyle and medication recommendations. In addition, efforts to understand how positive reporting bias and social desirability may contribute to these scores remains an important area of future investigation. Strategies to obtain a wider spread of responses are recommended, such as adjusting the wording or response categories and range.

When we analyzed scale mean scores according to demographic criteria, we observed that black patients reported higher levels of perceived autonomy support in two of the four mean scale scores. This may suggest that either physicians communicate more effectively with black patients, or that black patients have different expectations regarding their physicians’ communication. There also appeared to be a trend toward an inverse relationship between the level of education and perceived autonomy support in three of the four scales, with more highly educated patients reporting lower mean scores. This suggests that patients with more education might be more assertive and expect more from their physicians. Overall, the study sample had relatively high levels of education, with 88 % having completed at least some college. How the scale may perform among a lower educated or lower literacy population should be examined.

We also observed that patients who underwent lumpectomy rated the autonomy supportiveness of their medical oncologist significantly higher than patients who underwent mastectomy, suggesting that medical oncologists might have a tendency to be more prescriptive in their conversations with patients who have undergone mastectomy. Studies have suggested that most patients who undergo mastectomy have the procedure due to either surgeon recommendation or an unsuccessful attempt at breast conserving surgery [26], both of which represent situations in which a patient would reasonably feel they had little decision-making autonomy. In the remaining patients who choose mastectomy due to preference, it is plausible that physicians may express disagreement with the patient’s choice, which would make those who chose mastectomy feel like they were in a less autonomy-supportive environment. Perceived autonomy support in patients who undergo mastectomy represents an area that merits additional examination in future work.

The mHCCQ assesses six dimensions of perceived autonomy support. It is possible that other domains not measured here may also play an important role in encouraging autonomous motivation. In previous studies, the mHCCQ has often been paired with other measures that assess autonomous self-regulation, such as the General Causality Orientation Scale, the Treatment Self-Regulation Questionnaire, and the Perceived Competence Scale [9, 11, 17, 27]. Inclusion of these scales may provide a more comprehensive and perhaps multi-dimensional measure of autonomy support and patient motivation for self-regulated behavior, though at the cost of a significantly longer survey. We chose to limit our evaluation to these six dimensions to keep our scales brief.

## Conclusion

In summary, our results represent the first use of the mHCCQ in cancer patients, and demonstrate that patients perceive and report differences in communication across provider types. Ratings of the overall treatment experience account for only half of the variance, suggesting that patients differentiate their ratings of autonomy support according to each provider, rather than lumping their experience into an overall rating. The psychometric properties of the six item mHCCQ suggest that it may be a useful tool in understanding patients’ perceptions of how well physicians facilitate autonomous motivation and self-regulated behavior, and like other subjective rating scales, these items may be considered inherently valid as they draw upon patient opinion and feeling. More expansive exploration regarding the validity of the mHCCQ in breast cancer patients, however, is still warranted. This could include examining how autonomy support may be associated with decision quality and personality attributes such as social desirability and optimism, as well as outcomes such as quality of life and cancer-related anxiety.

## Abbreviations

mHCCQ: Modified *Health Care Climate Questionnaire*
EFA: Exploratory factor analysis
BECCI: Behavior change counseling index
AJCC: American joint committee on cancer
MSKCC: Memorial sloan kettering cancer center
